# MOSFE-Capacitor Silicon Carbide-Based Hydrogen Gas Sensors

**DOI:** 10.3390/s23073760

**Published:** 2023-04-05

**Authors:** Artur Litvinov, Maya Etrekova, Boris Podlepetsky, Nikolay Samotaev, Konstantin Oblov, Alexey Afanasyev, Vladimir Ilyin

**Affiliations:** 1 Micro- and Nanoelectronics Department, National Research Nuclear University MEPhI (Moscow Engineering Physics Institute), Kashirskoe Highway 31, 115409 Moscow, Russia; 2 Engineering Center of Microtechnology and Diagnostics, St. Petersburg Electrotechnical University (ETU «LETI»), Professora Popova str. 5, 197022 St. Petersburg, Russia

**Keywords:** field-effect gas sensor, high-temperature ceramic package, capacitance–voltage characteristic, pulsed laser deposition, silicon technology, gas analysis

## Abstract

The features of the wide band gap SiC semiconductor use in the capacitive MOSFE sensors’ structure in terms of the hydrogen gas sensitivity effect, the response speed, and the measuring signals’ optimal parameters are studied. Sensors in a high-temperature ceramic housing with the Me/Ta_2_O_5_/SiC^n+^/4H-SiC structures and two types of gas-sensitive electrodes were made: Palladium and Platinum. The effectiveness of using Platinum as an alternative to Palladium in the MOSFE-Capacitor (MOSFEC) gas sensors’ high-temperature design is evaluated. It is shown that, compared with Silicon, the use of Silicon Carbide increases the response rate, while maintaining the sensors’ high hydrogen sensitivity. The operating temperature and test signal frequency influence for measuring the sensor’s capacitance on the sensitivity to H_2_ have been studied.

## 1. Introduction

Field-effect solid-state gas sensors based on metal–insulator–semiconductor (MIS) or metal–oxide–semiconductor (MOS) structures have been known for half a century. The beginning of the devices’ practical implementation was initiated by hydrogen-sensitive transistors based on Pd/SiO_2_/Si structures [1,2]. The structure type choice was largely due to the compatibility of the sensitive elements’ manufacturing process with silicon technology production [3]. This contributed to the sensor miniaturization and the acceptable parameter’s reproducibility in mass production. It is interesting to note that the first transistors in the early 1950s were made not from Silicon, but from Germanium, which has higher electrons’ and holes’ mobility, and which was easier to clean from impurities. However, over time, Germanium revealed a significant drawback that limited its further use. The band gap of Ge is only 0.67 eV, and, as a result, at a temperature of about 75 °C and above, Germanium transistors are inoperable due to the free excess electrons. The way out of this situation was to use Silicon with a band gap of 1.1 eV and the impurity Si wafers obtained by using gaseous diffusion technology. However, by the 1990s, Silicon had also approached the limits of its use, and the further electronics evolution required the accelerated development of the wide band gap (WBG) semiconductor technology [4,5]. Scientific and practical results of such studies are also successfully applied in the semiconductor gas sensors’ production [6,7,8,9,10,11,12].

Energy, transport, and various industries are the main areas of hydrogen sensor use at present and in the future. Hydrogen is used in metal smelting, household chemicals, glass manufacturing, semiconductor manufacturing, and oil extraction. Hydrogen is also used as a fuel for environmentally friendly cars. In addition, since Hydrogen is explosive, it is necessary to control its concentration in coal mines, nuclear reactors, battery rooms, etc. Therefore, the Hydrogen gas analyzers’ development based on explosion-proof sensors does not stop; nor does the improvement of their parameters in terms of speed and measurement accuracy [13,14].

**Figure 1:**
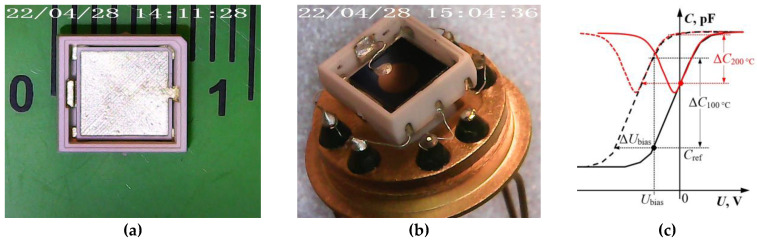
High-temperature ceramic package (without MOS structure) created using laser micromilling technology with Platinum and gold metallization (**a**). An example of the MOSFEC sensors’ high-temperature design: the standard TO-8 package is used as a base (adapter) for the monolithic sintered ceramic housing (**b**). An example of the CV characteristics’ shift of the Si-based MOS capacitor under the Hydrogen action at the operating temperature of 100 and 200 °C—black and red curves, respectively (**c**). Solid lines—CVC in the absence of H_2_, dotted lines—CVC in the presence of H_2_. See the text for a description of the designations.

This article investigates capacitive MOSFE hydrogen sensors in a high-temperature ceramic design (Figure 1a,b), the description of which is considered in detail in [15]. The physical basis for the capacitive MOSFE sensors’ operation is the field effect, which changes free charge carriers’ concentration in the semiconductor’s near-surface region at the interface with the insulator under the action of an electrical voltage applied to the sensor. When the sensor is exposed to the detected gas, its molecules diffuse through the electrode film to the metal–insulator interface, where they are adsorbed by active capture centers. This leads to a change in the electric field in the insulator and the semiconductor and a free charge carriers’ redistribution in the semiconductor’s near-surface region and, as a result, a shift in the MOS structure’s capacitance–voltage characteristic (CVC or CV characteristics) along the voltage axis (Figure 1c). The shift ΔU_bias_ value can be compared quantitatively with the detected gas concentration, while the MOSFE capacitor’s useful signal can be either directly the ΔU_bias_ value at a fixed reference capacitance C_ref_ value, or the change in capacitance ΔC at a fixed U_bias_ value.

Our technology-distinctive feature to produce MOSFEC sensors with the Pd/Ta_2_O_5_/SiO_2_/Si type structure is high gas sensitivity in the operating temperature range from 50 °C to 150 °C with a limit of detection (LOD) for Hydrogen at the level of 150 ppb [16,17]. Such a sensitivity, in our opinion, is largely due to a combination of the following technological factors: the use of Pulsed Laser Deposition (PLD) for the thin films’ fabrication; the capacitor type of the MOS structures; and a porous electrode with a diameter of 2 to 3 mm made of catalytically active Palladium. For comparison, [13] presents a large-scale review of semiconductor hydrogen sensors (resistive, based on Schottky diodes, MOS transistors, MOS capacitors, etc.) manufactured using various physical methods: Sol-Gel Annealing, Magnetron Sputtering (MS), Thermal Oxidation, Spray Pyrolysis, PLD, etc. The LOD of the most sensitive samples presented in this review is 5 ppm. In [18], the chemo-resistive nanocomposite NiO:Pd sensor capable of detecting Hydrogen concentrations in the air up to 300 ppb and operating in the temperature range of 115–145 °C was described. In [19], the best 50 ppb Hydrogen LOD result that we could find in the literature is presented: a gas sensor based on SnO_2_-loaded ZnO nanofibers fabricated using an electrospinning technique with optimal working temperature of 300 °C.

Nevertheless, exceptionally high sensitivity is not sufficient, and the main problem— which is the motivation for this work—is the following. The operating temperature of 50–150 °C, which is typical for MOSFEC Si-based sensors, gives a response speed of 5–10 min when detecting hydrogen concentrations at the units-hundreds of ppm level. This corresponds to other authors’ results for different types of hydrogen sensors [13,20,21]. Such indicators are not always acceptable for safety tasks in conditions where there is a risk of harmful and dangerous gases’ rapid formation and accumulation. Increasing operating temperatures can be a solution. For example, in review [13], the best response times to Hydrogen of 1000–1500 ppm are a few units to tens of seconds for sensors with an operating temperature of 300–500 °C.

At 200 °C and above, however, active generation of the intrinsic charge carriers occur in the Si semiconductor. As a result, the CV characteristics’ shape of classical Pd/SiO_2_/Si type MOS structures is significantly deformed (Figure 1c) and the ΔU_bias_ or ΔC values under the gas action measurement error increase, which worsens the MOSFEC sensors’ LOD parameter. In addition, a thin-film Pd electrode, which, according to our experimental data, begins to oxidize at 220 °C and loses its conductive properties, can also cause a failure in operation. A well-known solution to this problem is the WBG semiconductor use (for example, SiC, AlN, GaN, AlGaN, diamond) as a substrate, and catalysts resistant to high temperatures as a gate material; for example, Platinum or Ruthenium [8,22,23].

In this work, we used the SiC semiconductor, which has advantages such as high chemical inertness, physical stability, and high thermal conductivity [24]. All this makes the SiC product suitable for use in harsh environments such as high temperatures and radiation. For example, it was shown in [22] that MOS capacitors with the Pt/TaO_x_/SiO_2_/SiC structure (Pt is a porous electrode; n-type (0001) Si-face 4H-SiC substrates) can operate at the temperature of 200 °C in the environments with an extremely high concentration of water vapor (about 45% vol.). At the same time, they maintain sensitivity to H_2_, CO, ethane, and ethene with a LOD of a few units to tens ppm, which is applicable to solve the monitoring exhaust problem of the cell gases fuel based on Hydrogen or Hydrocarbons.

The aim of this work is to create MOSFEC sensors using PLD technology based on a SiC semiconductor substrate to expand the operating temperature range, increase speed and maintain high sensitivity to Hydrogen with a LOD of at least 150 ppb, as well as to compare the characteristics of the obtained sensors with classical sensors on the Si substrate.

## 2. Experimental

### 2.1. Samples’ Production and Setup Description

**Figure 2:**
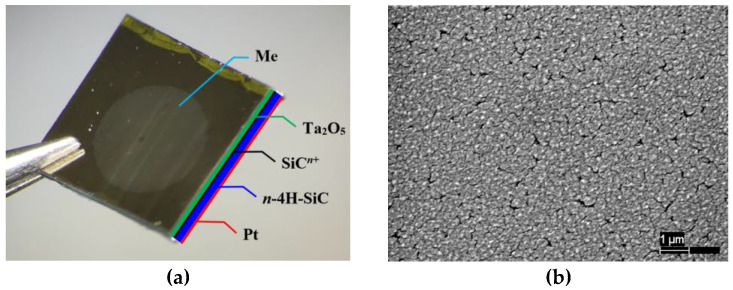
The MOS structure’s scheme used in this work: the sequence of layers from top to bottom Me/Ta_2_O_5_/SiC^n+^/4H-SiC/Pt, where “Me” is the upper metal gate film with a diameter of 2.5 mm (**a**). SEM photograph of the palladium electrode porous surface (**b**).

For research, two kinds of the Me/Ta_2_O_5_/SiC^n+^/4H-SiC/Pt type structures (hereinafter SiC samples) were fabricated (Figure 2a), where the following are uniform:

- SiC^n+^/4H-SiC substrate (*n*-type epitaxial layer 4 µm thick with uncompensated donors’ concentration N_d_-N_a_ = 6…7·10^15^ cm^−3^, and *n*-4H-SiC substrate with resistivity 0.015…0.017 Ω·cm),
- Ta_2_O_5_ layer obtained by Tantalum deposition using PLD and subsequent metal film oxidation in the air by using a muffle furnace at 650 °C,
- ohmic Pt contact, also obtained by the PLD method.

The metal electrode (gate) film “Me” is a distinctive feature: for Samples No. 1—Palladium obtained by the PLD method; for Samples No. 2—Platinum formed by MS on the surface of an insulator doped with Palladium by the PLD. 

Palladium doping was used to maintain the sensitivity at the same level as that of sensors with a Pd/Ta_2_O_5_ metal–insulator interface, which largely determines the gas-sensitive properties of sensors with a porous electrode [25,26]. For example, Figure 2b shows the Pd electrode surfaces SEM photograph, which illustrates the metal film porosity. Control samples of Si-based MOS structures for gas sensors were used as a “starting point” for comparing and identifying the WBG semiconductor contribution. The control samples were fabricated based on a *n*-type silicon wafer (resistivity 15 Ω·cm) with a basic thermally oxidized silicon insulator layer.

A solid-state yttrium aluminum garnet laser was used in the PLD setup. The deposition was carried out at a pressure of 1 × 10^−5^ Torr. The MS system was equipped with a 3-inch circular planar magnetron (Pinch Magneto series). The magnetron was operated at an argon pressure of 1 Pa (7.5 × 10^−3^ Torr). The target material was deposited on the substrate surface through a ceramic mask. Both methods of thin films’ vacuum deposition (which have been used since the 1960s–1970s, are well studied, and debugged) provide high adhesion of the deposited film to the substrate due to the optimal value of the deposited particles’ energy without damaging the substrate surface, and without mutual mixing of the target and substrate materials. This makes it possible to increase the yield of high-quality and long-term stable MOS structures and to achieve a minimum spread in the characteristics of gas sensors based on them [16,27].

On the obtained MOS structures’ bases, gas sensors were fabricated in specialized miniature metal-ceramic packages measuring 6.0 × 6.4 × 2.0 mm from monolithic 96% aluminum oxide ceramics with a built-in platinum heater using Adaptive Laser Microengraving technology [15,28,29,30]. This technology of laser processing of monolithic sintered ceramics is an affordable alternative to LTCC technology (low-temperature co-fired ceramics) and allows us to create metal-ceramic packages quickly and cheaply in small-scale production using non-standard solutions. An example of the sensors’ constructive implementation is shown in Figure 1b. A similar ceramic housing, in contrast to the usual glass-to-metal one made (operating temperature limit 250 °C)—which is also shown in Figure 1b, and used as a carrier and a DIP adapter—provides the ability to operate at temperatures up to 500 °C with power consumption of 0.5 W at 200 °C.

The studies were carried out on an experimental setup in which gas concentrations were created by the static dilution method of Hydrogen with the air. To do this, MOSFEC sensors, the operating temperature of which was maintained and regulated by an electronic board, were placed in a sealed fixed volume chamber with the possibility of pumping and updating the gas mixture with the pump. It was also possible to dose Hydrogen, obtained from the generator by the electrolysis method or control gas mixture cylinder, into the volume of the chamber using a measuring syringe.

To measure the sensors’ response under the Hydrogen action, two measuring devices were used independently of each other: (1) electronic circuit board based on the PCap-01D chip (further in the text “Board CDC”) [31,32] and (2) precision digital meter RCL Aktakom AMM-3068 (NPP “ELIKS” company, Moscow, Russian Federation [33]) with the following settings: test frequencies of the measuring signal in the range from 2 to 200 kHz; test signal voltage is fixed at 50 mV; output impedance 10 Ω; and scanning speed 2.7 meas./s. 

The electronic Board CDC used includes the following functional blocks: (1) capacitance conversion; (2) bias voltage generation; (3) heating and sensor’s temperature control; and (4) communication with external devices and control. The operation principle is since the converter periodically charges and discharges the MOS capacitor and determines the capacitance value by the discharge time (which is uniquely related to the capacitance value). The measurement upper limit was 3500 pF. The bias voltage in the range from −4 to +0.5 V is set by the microcontroller and the DAC chip. The sensor’s operating temperature is set and regulated according to a proportional-integral algorithm using a program stored in the microcontroller’s memory. The sensor temperature is measured by a thermistor also connected to the circuit. The voltage from the thermistor, proportional to the sensor’s temperature, is digitized by the ADC chip, and read by the microcontroller via the SPI interface. 

### 2.2. Response Speed Determination

**Figure 3:**
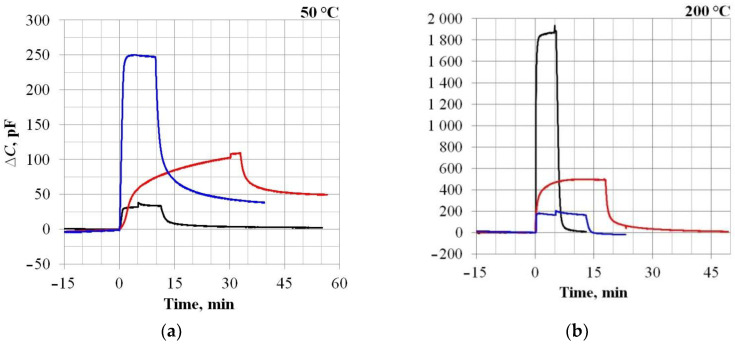
An example of the sensors’ dynamic characteristics exposed to a 1000 ppm H_2_ at different operating temperatures: 50 °C (**a**) and 200 °C (**b**). The beginning of the gas concentration supply corresponds to zero on the abscissa. The gas feed duration varied depending on the readings’ stabilization rate. Black lines—Sample No. 1; red lines—Sample No. 2; and blue lines—Control sample.

**Figure 4:**
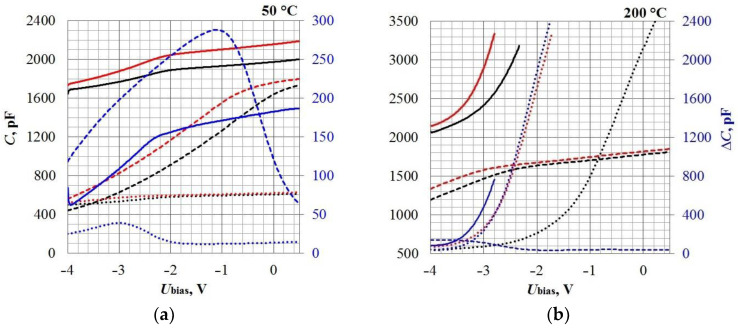
Sensors’ CV characteristics in the air (black lines) and under the act of 1000 ppm H_2_ (red lines) at different operating temperatures: 50 °C (**a**) and 200 °C (**b**), taken by using Board CDC. The blue lines indicate the sensors’ responses ΔC(U_bias_) corresponding to the CV characteristics’ shift and plotted on the auxiliary axis. Dotted lines—Sample No. 1; solid lines—Sample No. 2; and dash lines—Control sample.

The tasks of the first experiment series were to determine the sensors’ response speed and to assess the sensitivity level using the Board CDC, which, in addition to measuring the capacitance value of the sensor, makes it possible to measure the CV characteristics. The results are shown in Figure 3 and Figure 4 and in Table 1. 

The results predictably show that, as the temperature increases, the response times of all samples improve significantly. In this case, the sensitivity of the Control sample (Si) decreases, while that of the SiC samples, on the contrary, increases significantly. According to the response times, the Control sample and Sample No. 1 (both with Palladium electrodes) are close to each other. Sample No. 2 with a Platinum electrode is noticeably worse, both at 50 °C and at 200 °C. On Figure 4 shows the sensor capacitance dependencies (along the main Y-axis) and the response value ∆C (along the auxiliary Y-axis), when Hydrogen is supplied, on the bias voltage U_bias_ applied to the MOS capacitor’s plates. This dependence is non-linear, so the sensors’ sensitivity level, estimated using the Board CDC and quantified in Table 1, is relative. For a more correct comparison of the sensitivity parameters, it is necessary to turn to the physical foundations of the capacitive MOS structures’ gas sensitivity and consider the magnitude of the shift in the CV characteristics ΔU_bias_ under the gas action. However, due to the peculiarities of the Board CDC’s measuring circuit, the value of ΔU_bias_ depends on the reference capacitance value C_ref_, by analogy with the dependence ΔC(U_bias_): see Figure 4. In addition, at 200 °C, the SiC sensors’ capacity, as can be seen from Figure 4b, at some values, U_bias_ exceeds 3500 pF, which is beyond the Board CDC’s measurement capabilities. For these reasons, further sensitivity studies were carried out by using the RCL-meter, taking into account previously obtained information about the sensor’s response speed.

### 2.3. The Influence of the Measuring Test Signal Frequency

**Figure 5:**
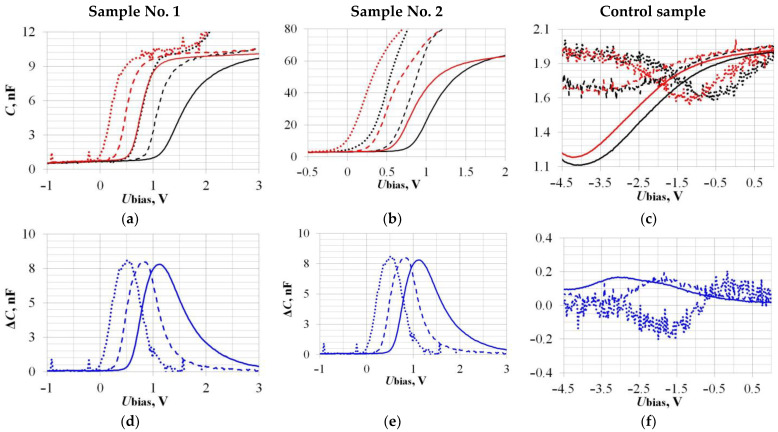
Influence of the measuring test signal frequency on the CV characteristics’ shift under the Hydrogen action (**a**–**c**). Black lines—CV characteristics in the absence of H_2_; red lines—under the act of 1000 ppm H_2_; Dotted lines—2 kHz, dash lines—20 kHz and solid lines—200 kHz. Dependencies of the capacitive sensors’ responses ΔC(U_bias_) on the measuring signal frequency, corresponding to the CV characteristics’ shift: Sample No. 1 (**d**), Sample No. 2 (**e**), and Control sample (**f**).

Before the main study of the sensors’ hydrogen sensitivity, the effect of the measuring test signal frequency influence was established. The CV characteristics’ shift at the sensors’ operating temperature of 170 °C was studied. The experimental frequencies’ values of the measuring signal with a fixed amplitude of 50 mV were 2, 20, and 200 kHz. The results are shown in Figure 5 and in Table 2.

As can be seen, the Control Si samples’ CV characteristics (Figure 5c,f) are greatly different from the others SiC Samples No. 1 and No. 2. The main reason for this is that the operating temperature of 170 °C is already high enough to start the intrinsic charge carriers’ generation process in Si samples, and this deforms the CV characteristics and increases the useful signal’s measurement error. We have already talked about this in the Introduction (Figure 1c) and confirmed it experimentally here.

It has been shown that, within the measurement error, the values of ΔU_bias_ do not depend on the measuring test signal frequency, which correlates with our previous results [32,34]. The value of ΔC_max_ in the case of Sample No. 2 decreases non-linearly as the test signal frequency increases (Figure 5e). Further, in the work, the measuring signal parameters were used: 20 kHz, 50 mV.

### 2.4. The Influence of the Sensor Operation Temperature

**Figure 6:**
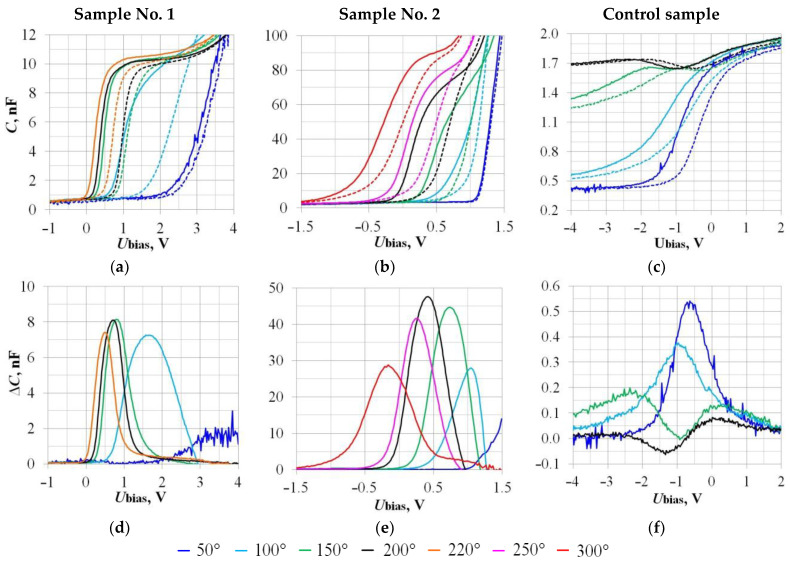
Influence of the sensor operating temperature on the CV characteristics’ shift under the Hydrogen action (**a**–**c**). Dashed lines—CV characteristics in the absence of H_2_; solid lines—under the act of 1000 ppm H_2_. Dependencies of the capacitive sensors’ responses ΔC(U_bias_) on the operating temperature, corresponding to the CV characteristics’ shift (**d**–**f**).

The results of the sensor operating temperature influence study on the hydrogen sensitivity are shown in Figure 6 and in Table 3. For comparison, Table 3 also shows the dependence ΔC_max_(U_bias_) data obtained on the Board CDC (Figure 4). The total range of temperatures studied was from 50 to 300 °C.

As expected, the SiC samples exhibited a lower temperature dependence compared to the Si samples. For example, the Control sample’s CVC is deformed starting from 150 °C. At 200 °C, the ΔC(U_bias_) function becomes sign-variable, which is unacceptable for a sensor gas analyzer. In this case, with a response to 1000 ppm H_2_, there are U_bias_ values at which ∆C < 0; for example, if U_bias_ = −1.5 V, then ∆C = −0.05 nF: see Figure 6f. This means that the gas analyzer will register a decrease signal in the hydrogen concentration, although in fact the opposite is true. Similar problems may arise in the case of the Si samples with a high operating temperature and when measuring the useful signal as ΔU_bias_(C_ref_). 

When comparing the SiC samples with different electrodes (Pd and Pt), we note the following features. The sensors’ operating temperature—at which it is possible to register the maximum value of the response ΔC_max_ per 1000 ppm H_2—_for both samples is 150–200 °C. Therefore, the use of Platinum for high-temperature performance is not a necessary condition for MOSFEC hydrogen sensors. Palladium remains the decree that provides the highest sensitivity: compare the ΔU_bias_ values for Samples No. 1 and No. 2 in Table 3. In the operating temperature range of 50–150 °C, Si samples are more efficient than SiC.

Thus, the following optimal operating temperatures for sensors are set.

- 200 °C for SiC Samples No. 1 and No. 2;
- 100 °C for the Control Si sample.

The choice is due to the balance between sensitivity and speed. These values were used further in the study of the Hydrogen sensitivity in the concentration range from 1 to 1000 ppm.

### 2.5. Investigation of the Hydrogen Sensitivity and LOD

As noted in the Introduction, the MOSFEC sensor’s useful signal (and, therefore, the sensitivity) can be measured in two ways: by the ΔU_bias_ value at a fixed reference capacitance C_ref_ value, or by the change in capacitance ΔC at a fixed U_bias_ value (Figure 1c). 

However, experimental data have shown that the sensitivity estimate can be highly dependent on the measurement method and measurement signal parameters: compare the data obtained with the RLC-Meter and the Board CDC data (Table 3). The operation principle of the capacitive MOSFE gas sensors and the experimental results presented in Section 2.3 and Section 2.4 indicate that, for a correct comparative analysis of different samples, it is necessary to focus on the initial signal ΔU_bias_. However, the mediated ΔC signal has a higher resolution, and it is more efficient for estimating LOD.

**Figure 7:**
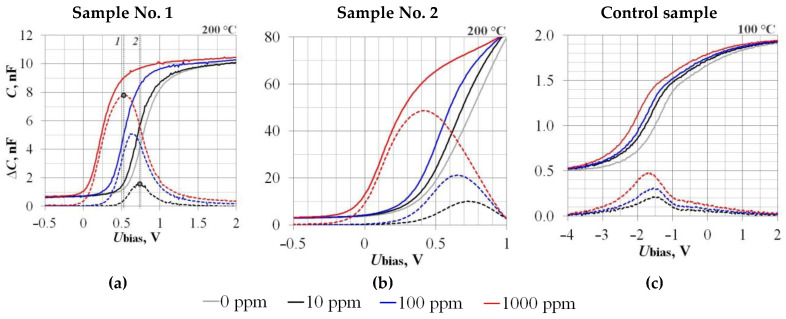
The experimental samples’ hydrogen sensitivity calibration: Sample No.1 (**a**), Sample No.2 (**b**), and Control sample (**c**). Lines *1* and *2* (**a**) show the U_bias_ value influence on the recorded signal ΔC value at different hydrogen concentrations (see explanations in Section 3).

**Figure 8:**
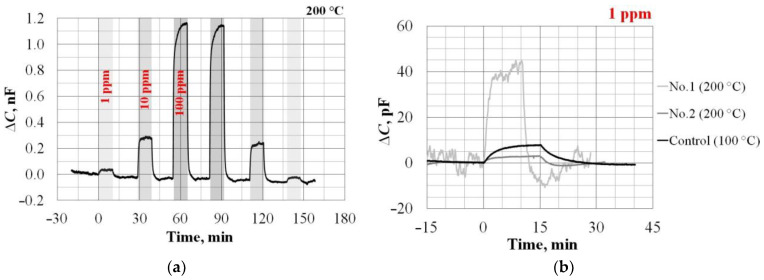
Response dynamics of the Sample No. 1 to different concentrations of Hydrogen, obtained using the Board CDC (**a**). Comparison of the sensors’ different type responses to 1 ppm Hydrogen (**b**).

The calibration results of the sensors’ sensitivity to Hydrogen according to the RLC-Meter data are presented in Figure 7 and in Table 4, and according to the Board CDC data on the example of Sample No. 1 in Figure 8.

**Figure 9:**
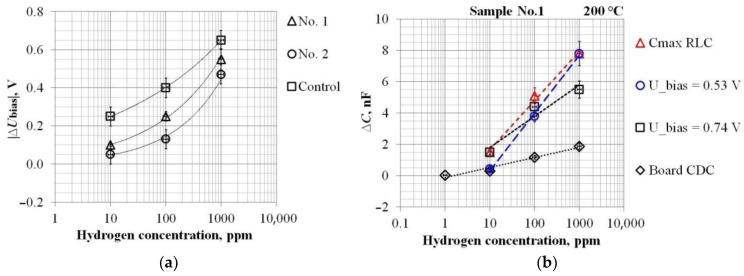
The sensors’ calibration characteristics ΔU_bias_(C_H2_) (**a**). The calibration characteristics ΔC(C_H2_) of Sample No. 1 at the different measuring circuit operating settings and comparison with the experimental Board CDC data (**b**).

According to the ΔU_bias_ data in Table 4, the leaders in Hydrogen sensitivity are Si samples with an operating temperature of 100 °C (Figure 9a). However, the difference between Si and SiC samples is not so great. This can be explained by the metal–insulator interface uniformity, which largely affects the sensitivity and function ΔU_bias_(C_H2_). However, according to the ΔC_max_ data, the Hydrogen sensitivity of SiC samples with an operating temperature of 200 °C is 1–2 orders of magnitude higher than that of Si. From our point of view, the main reason for this is the lower temperature dependence of the SiC samples’ CV characteristics.

According to the data in Figure 8b, we calculate the LOD of Hydrogen for different samples:

LOD = (3 × *N*)/S_max_ = (3 × *N*) × (C_H2_/ΔC_max_),

where *N* is the noise of the sensor capacitance signal, pF; S_max_ is the maximum sensor sensitivity, pF/ppm; and ΔC_max_ is the maximum change in sensor capacitance, pF, under the action of H_2_ with C_H2_ concentration, ppm. For example, for the Control sample, the calculated LOD would be:

LOD _Control sample_ = (3 × 0.2) × (1/8) = 75 ppb.

By analogy, for the remaining samples we will have:

- LOD Sample No. 1–375 ppb;
- LOD Sample No. 2–500 ppb;

Response times at 1–100 ppm of Hydrogen, min;

- Sample No. 1—τ_0.9_ = 1 ± 0.5; τ_0.1_ = 2 ± 1; τ_full_ = 7 ± 2;
- Sample No. 2—τ_0.9_ = 5 ± 1; τ_0.1_ = 5 ± 2; τ_full_ = 30 ± 10;
- Control sample—τ_0.9_ = 5 ± 3; τ_0.1_ = 10 ± 5; τ_full_ = 20 ± 5.

Therefore, using the data obtained, we will answer the main questions of this work: What are the advantages of using SiC and what potential difficulties are associated with it?

## 3. Discussion

Let us turn again to Figure 9, which shows the calibration data for the sensors’ hydrogen sensitivity. Figure 9a, already mentioned above, shows that, in terms of increasing the signal ΔU_bias_, the use of SiC does not bring benefits compared to Si. However, in this work, the main goal is to accelerate the response speed of the sensor by increasing the operating temperature. This was achieved due to Sample No. 1 with a Pd electrode. Nevertheless, it was shown that the sensitivity can also be increased by registering the useful signal not by the value of ΔU_bias_, but by ΔC (Figure 7, Table 4). This approach has some peculiarities. Figure 9b, using the example of Sample No. 1, illustrates the calibration characteristic ΔC(C_H2_)’s dependence on the measuring device (RLC-meter or Board CDC) and the operating settings choice. The “C_max_” curve corresponds to ideal conditions under which the maximum sensor sensitivity is achieved over the entire range of gas concentrations, but, in reality, this is unattainable. Examples of really possible calibration characteristics are the curves corresponding to U_bias_ = 0.53 V or U_bias_ = 0.74 V (see also lines *1* and *2* in Figure 7a). As can be seen from Figure 9b, such calibrations will differ from ideal ones to the measurements’ detriment of either high or low Hydrogen concentrations. Unfortunately, this feature is equally inherent for all experimental samples (Table 4).

Even in the case of measuring ΔC, however, the higher sensitivity of SiC samples did not contribute to better LOD values. This can be explained by the fact that Board CDC is a circuit solution designed for the Si-based sensors. Therefore, in order to record the ΔC signal in the case of SiC samples as efficiently as possible, optimization is required.

Thus, for the MOSFEC hydrogen sensors in the substitution from the Si substrate to SiC with the preservation of the Pd electrode, it was possible to achieve the following:

(1) reducing the temperature effect on the CV characteristics;
(2) increase of Hydrogen sensitivity function ΔC(C_H2_) by 1–2 orders of magnitude;
(3) the response speed increase is not worse than 2 times.

In the future, SiC will also make it possible to miniaturize the MOSFEC sensor while maintaining a high sensitivity level. Therefore, in [35,36], one of such methods is described, which consists of the nanocomposite Pd:SnO_2_ film formation obtained using the Reactive Sputtering. In this case, the sensor operation efficiency requires an operating temperature increase in order to activate the oxygen vacancies’ (adsorption centers) formation on the SnO_2_ crystallites surface. 

Since the idea of upgrading field-effect gas sensors by using WBG semiconductors is not new, it is interesting to compare our results with other authors’ data. Thus, in [21], using the example of hydrogen sensors based on Schottky diodes and Pt/Ta_2_O_5_ structures deposited on different Si and SiC substrates by radio frequency (RF) sputtering, a comparison of the sensors’ operating parameters at temperatures from 25 °C to 200 °C was undertaken. The authors tested samples for exposure to H_2_ with a concentration in the range from 600 ppm to 1% vol. (10,000 ppm) and showed that the SiC sensor exhibits relatively greater sensitivity, and the Si sensor a faster response. For example, at an operating temperature of 150 °C, the characteristic response times to 1250 ppm Hydrogen for Si samples were about τ_0.9_ = 2 min and τ_0.1_ = 6 min, and for SiC samples, they were τ_0.9_ = 5 min and τ_0.1_ = 13 min. Comparing our results (Figure 3, Table 1) with [21], we see that the response times of the Schottky diodes with a Pt contact are comparable to SiC Sample No. 2 with the Pt electrode, while our sensors with the Pd electrode (both Si-based and SiC-based) at 200 °C have a response time of 30 to 70 s. 

Table 5 presents the sensitivity assessing results of the experimental samples studied in this work by the value of ΔU_bias_ in comparison with the voltage shift data of the other authors. The comparison shows that capacitive MOSFE sensors—both ours and those of other colleagues—are more sensitive to hydrogen.

It is worth noting, however, the inevitable difficulties and limitations associated with the use of the SiC-based sensors at high operating temperatures. For example, according to estimates [37], SiC single crystals are capable of operating at 500 °C and higher, but most of the other typical structural gas sensors’ elements (housing, metal contacts, etc.) are either not able to withstand such temperatures for a long time or have a strong limited resource. This is due to several high temperature undesirable consequences: materials’ mutual diffusion, thermal expansion, corrosion, etc. Thus, the WBG semiconductors’ use imposes stringent requirements on the gas sensors’ design; therefore, it took more than 25 years for their commercial implementation [10,12,38]. Besides, high temperature increases the catalytic processes efficiency on the electrode surface and thus worsens the sensors’ selectivity parameters. For example, in [39], to control SO_2_ in emission desulfation systems in the energy sector, the authors propose to combat non-selectivity, i.e., reduce the O_2_, CO, and NO_x_ influence by using the cyclic mode of the sensor heating up to 350–400 °C (to shift the maximum sensitivity in SO_2_ favor) and carrying out linear discriminant analysis (LDA). 

**Table 5:** The comparison of the experimental samples’ parameters studied in this work with the other authors’ results.

Structure Type	Gas Sensor Type	Operating Temperature, °C	Hydrogen Concentration, ppm	Voltage Shift, mV	References
Pt/nanostructured RuO_2_/SiC	Schottky Diode	240	600	57	[9]
Pt/SnO_2_ nanowires/SiC	Schottky Diode	420	2500	70	[40]
			10,000	132	
		530	2500	95	
			10,000	310	
Pt/WO_3_/SiC		530	10,000	80	
Pt/Ga_2_O_3_/SiC		310	10,000	210	
Pt/TaO_x_/SiO_2_/SiC	MOSFEC	300	500	325	[22]
Pd/Ta_2_O_5_/SiO_2_/Si	MOSFEC	100	500	470	[34]
Pd/Ta_2_O_5_/SiO_2_/Si (Control Sample)	MOSFEC	100	100	400	this work
			1000	650	
Pd/Ta_2_O_5_/SiC (Samples No. 1)		200	100	250	
			1000	550	
Pt/Ta_2_O_5_/SiC (Samples No. 2)			100	130	
			1000	450	

## 4. Summary

MOSFEC hydrogen sensors in a high-temperature ceramic housing based on Me/Ta_2_O_5_/SiC^n+^/4H-SiC/Pt structures type with two types of gas-sensing electrodes were fabricated: Palladium, obtained using PLD; and Platinum formed using MS. The features of the wide band gap SiC semiconductor use in the capacitive MOSFE sensors’ structure in terms of the Hydrogen gas sensitivity effect, the response speed, and the measuring signals’ optimal parameters were studied. The operating temperature and test signal frequency influence for measuring the sensors’ capacitance on the sensitivity to H_2_ were studied. It has been experimentally established that the sensors’ operating temperature, at which it is possible to register the maximum value of the response to H_2_, for SiC-based samples both with a Palladium electrode and Platinum, is 150–200 °C. In the operating temperature range of 50–150 °C, Si samples are more efficient than SiC. The calculated LOD_H2_ values for the experimental samples range from 75 to 500 ppb. It is shown that with the MOSFEC Hydrogen sensors in the substitution from the Si substrate to SiC with the preservation of the Pd electrode, it was possible to achieve a reduction the temperature effect on the CV characteristics, an increase of Hydrogen sensitivity function ΔC(C_H2_) by 1–2 orders of magnitude, and the response speed increase is not worse than 2 times. In the future, SiC will also make it possible to miniaturize the MOSFEC sensor while maintaining a high sensitivity level.

## Figures and Tables

**Table 1:** Sensors’ response times under the act of 1000 ppm H_2_ and relative sensitivity level corresponding to the U_bias_ value received by using the Board CDC.

Sample	T, °C	τ_0,9_, min	τ_0,1_, min	τ_full_, min	Sensitivity, pF/ppm	U_bias_, V
**No. 1**	50	1	≈20	>45	0.04	−3.5
	200	0.5	1.2	≈10	1.88	−2.0
**No. 2**	50	20	∞	∞	0.11	−3.0
	200	4	7	≈45	0.50	−3.0
**Control**	50	1	>30	∞	0.25	−2.0
	200	0.5	1	3	0.16	−3.5

Designations: **T**—sensor operating temperature; **τ_0.9_**—time during which the sensor response to Hydrogen supply reaches 90% of the maximum value; **τ_0.1_**—time required for the sensor readings to return to the 10% level of the maximum response value after Hydrogen removal; **τ_full_**—time required to sensor readings’ return to the zero level after the Hydrogen removal; **Sensitivity**—ratio of the response value ∆C to the supplied Hydrogen concentration value (ΔC/C_H2_); **U_bias_**—bias voltage value applied to the MOS structure, relative to which the response value ∆C is determined.

**Table 2:** Data of the measuring test signal frequency influence on the sensitivity to 1000 ppm H_2_ at the sensor operating temperature of 170 °C.

Parameter →		U_bias_, V	ΔC_max_, nF	C_ref_, nF	ΔU_bias_, V
Sample	↓ Frequency, kHz				
**No. 1**	**2**	+0.5	8.0 ± 1.5	4.5 ± 0.5	−0.58 ± 0.05
	**20**	+0.8	8.0 ± 1.0		−0.59 ± 0.05
	**200**	+1.1	7.8 ± 1.0		−0.70 ± 0.10
**No. 2**	**2**	+0.4	34 ± 3	29 ± 6	−0.25 ± 0.05
	**20**	+0.6	30 ± 3		−0.24 ± 0.05
	**200**	+0.9	22 ± 3		−0.23 ± 0.05
**Control**	**2**	0	0.11 ± 0.05	1.6 ± 0.2	−0.52 ± 0.25
	**20**	−1.9	0.16 ± 0.02		−0.50 ± 0.10
	**200**	−3.0	0.17 ± 0.01		−0.45 ± 0.05

Designations: **ΔC_max_**—maximum value of the sensor response under the act of Hydrogen, corresponding to the U_bias_ value indicated in the Table; **ΔU_bias_**—value of the sensor CV characteristic shift under the act of hydrogen, corresponding to the reference capacitance value **C_ref_** indicated in the Table. See Table 1 for other designations.

**Table 3:** Data of the sensor operating temperature influence on the sensitivity to 1000 ppm H_2_ depending on the measuring device.

Measuring Device →		RLC-Meter (20 kHz; 50 mV)				Board CDC	
Parameter →		U_bias_, V	ΔC_max_, nF	C_ref_, nF	ΔU_bias_, V	U_bias_, V	ΔC_max_, nF
Sample	↓ T, °C						
**No. 1**	**50**	≈+3.6	≈1.5	5.0	−0.20 ± 0.10	−3.0	0.04
	**100**	+1.7	7.3 ± 1.0		−1.20 ± 0.05	—	—
	**150**	+0.8	8.1 ± 1.0		−0.60 ± 0.05	—	—
	**200**	+0.8	8.0 ± 1.0		−0.60 ± 0.05	≥1.8	>2.4
	**220**	+0.5	7.4 ± 1.0		−0.50 ± 0.05	—	—
**No. 2**	**50**	≈+2.5	≈80 ± 20	30	→ 0	+0.5	0.19
	**100**	+1.1	35 ± 5		−0.20 ± 0.05	—	—
	**150**	+0.75	45 ± 1		−0.45 ± 0.05	—	—
	**200**	+0.46	47 ± 1		−0.44 ± 0.05	≥2.8	>0.75
	**250**	+0.27	41 ± 1		−0.35 ± 0.05	—	—
	**300**	−0.15	30 ± 1		−0.30 ± 0.05	—	—
**Control**	**50**	−0.7	0.52 ± 0.02	1.0	−0.55 ± 0.05	−1.1	0.29
	**100**	−1.0	0.37 ± 0.01		−0.67 ± 0.05	—	—
	**150**	−2.3	0.18 ± 0.01	1.7	−0.75 ± 0.05	—	—
	**200**	+0.1	0.08 ± 0.01		−0.50 ± 0.10	−4.0	0.15

See Table 2 for Designations.

**Table 4:** Data of the hydrogen concentration influence on the sensor response.

Parameter →		U_bias_, V	ΔC_max_, nF	C_ref_, nF	ΔU_bias_, V
Sample	↓ H_2_, ppm				
**No. 1**	**10**	+0.74	1.5	5.0	−0.10
	**100**	+0.63	5.1		−0.25
	**1000**	+0.53	7.8		−0.55
**No. 2**	**10**	+0.75	10.0	30.0	−0.05
	**100**	+0.65	21.2		−0.13
	**1000**	+0.42	48.6		−0.47
**Control**	**10**	−1.5	0.20	1.0	−0.25
	**100**	−1.6	0.31		−0.40
	**1000**	−1.7	0.48		−0.65

See Table 2 for Designations.

## Data Availability

Data sharing not applicable.
